# Systematic Analysis of Time-Series Gene Expression Data on Tumor Cell-Selective Apoptotic Responses to HDAC Inhibitors

**DOI:** 10.1155/2014/867289

**Published:** 2014-10-13

**Authors:** Yun-feng Qi, Yan-xin Huang, Yan Dong, Li-hua Zheng, Yong-li Bao, Lu-guo Sun, Yin Wu, Chun-lei Yu, Hong-yu Jiang, Yu-xin Li

**Affiliations:** ^1^National Engineering Laboratory for Druggable Gene and Protein Screening, Northeast Normal University, Changchun 130024, China; ^2^School of Computer Science and Information Technology, Northeast Normal University, Changchun 130117, China; ^3^Department of General Internal Medicine, First Hospital of Jilin University, Changchun 130021, China; ^4^Research Center of Agriculture and Medicine Gene Engineering of Ministry of Education, Northeast Normal University, Changchun 130024, China

## Abstract

SAHA (suberoylanilide hydroxamic acid or vorinostat) is the first nonselective histone deacetylase (HDAC) inhibitor approved by the US Food and Drug Administration (FDA). SAHA affects histone acetylation in chromatin and a variety of nonhistone substrates, thus influencing many cellular processes. In particularly, SAHA induces selective apoptosis of tumor cells, although the mechanism is not well understood. A series of microarray experiments was recently conducted to investigate tumor cell-selective proapoptotic transcriptional responses induced by SAHA. Based on that gene expression time series, we propose a novel framework for detailed analysis of the mechanism of tumor cell apoptosis selectively induced by SAHA. Our analyses indicated that SAHA selectively disrupted the DNA damage response, cell cycle, p53 expression, and mitochondrial integrity of tumor samples to induce selective tumor cell apoptosis. Our results suggest a possible regulation network. Our research extends the existing research.

## 1. Introduction

Histone acetylation is controlled by histone acetyltransferases (HATs), while histone deacetylases (HDACs) counterbalance activity of HATs [1]. HDAC activity is deregulated in tumor cells [2–6]. HDAC inhibitors are potent antiproliferative agents that cause tumor cell-selective apoptosis in both cell-based and clinical studies [7]. SAHA (suberoylanilide hydroxamic acid or vorinostat) was approved in October 2006 by the US Food and Drug Administration as the first nonselective HDAC inhibitor for treating cutaneous T-cell lymphoma [8]. SAHA affects histone acetylation in chromatin and a variety of nonhistone substrates, thus influencing many cellular processes [9]. In particular, SAHA mediates tumor cell-selective apoptosis in a time-dependent and concentration-dependent manner while leaving normal cells relatively unharmed [10–12]. However, the mechanism of SAHA is currently not well understood. Recently, Bolden et al. investigated tumor cell-selective, proapoptotic transcriptional responses induced by SAHA using time-series gene expression profiles [13]. More than 4200 genes responded to SAHA differently in normal and transformed cells by gene ontology (GO) [14] analyses with the DAVID tool [15]. Genes involved in induction of programmed cell death and apoptotic program were enriched in SAHA-treated transformed cells. Bcl-2 family genes were identified as the proapoptotic gene expression signature using the IPA tool (Ingenuity Systems, http://www.ingenuity.com/). These findings provide new insights into the transcriptional effects of HDAC inhibitors in normal and transformed cells and implicate specific molecules and pathways in the tumor-selective cytotoxic activity of SAHA. However, Bolden et al. identified only Bcl-2 family genes as the tumor cell-selective proapoptotic signature of SAHA. On the one hand, too few genes might meet the threshold for statistical significance because of modest differences in signals relative to the background noise. On the other hand, pathway analysis based on differently expressed genes might fail to detect biological processes across an entire network of genes including metabolic pathways, transcriptional programs, and stress responses, because changes in individual gene expression are sometimes subtle [16]. In this study, we propose a novel framework for detailed investigation of the mechanism of tumor cell apoptosis selectively induced by SAHA. Pathway gene expression coherence levels in tumor and normal cells treated with SAHA were systematically assessed using an improved *H*-score method in the context of predefined pathways and functional categories in the Kyoto Encyclopedia of Genes and Genomes (KEGG) [17], BioCarta [18], and GO [14]. The variation patterns of pathway gene expression coherence (TVPC) in the time series were examined. Significant gene expression profiles and profile pairs were enriched using STEM (short time-series expression miner) software [19]. Our analyses indicated that SAHA selectively disrupted the DNA damage response (DDR), cell cycle, p53 expression, and mitochondrial integrity of tumor samples to induce selective tumor cell apoptosis. Our results suggest a possible regulation network. Our research extends the original research of Bolden et al.

## 2. Materials and Methods

### 2.1. Time-Series Gene Expression Dataset

In the work of Bolden et al., time-series microarray experiments on normal (BJ) and transformed cells (BJ LTSTERas) determined gene expression with SAHA treatment [13]. Genomewide expression profiles were measured with Affymetrix GeneChip HG-U133 plus 2.0 arrays at the 4 h, 12 h, and 24 h time points. The gene expression data can be downloaded from NCBI Gene Expression Omnibus (GSE43010) (http://www.ncbi.nlm.nih.gov/geo/). Our experimental dataset for further analysis was from Bolden et al.

### 2.2. Pathway Definition

Well-annotated gene sets representing all biological processes are critical for meaningful and insightful interpretation of large-scale genomic data. Molecular Signatures Database (MSigDB) is one of the most widely used repositories of annotated genes sets involved in biochemical pathways (http://www.broadinstitute.org/gsea/msigdb/index.jsp) [20]. The three datasets MSigDB v4.0: c2.cp.kegg.v4.0 (KEGG), c2.cp.biocarta (BioCarta), and c5.all.v4.0 (GO) were used for our pathway gene analysis. We excluded gene sets with fewer than 15 genes or more than 500 genes because small or large gene sets might affect the veracity of calculations, according to GSEA (gene set enrichment analysis) [16]. We investigated a dataset of 1307 gene sets (176 KEGG gene sets, 139 BioCarta gene sets, and 992 GO gene sets).

### 2.3. Computational Method of Pathway Coherence and Significance

Huang et al. proposed the *H*-score method (Kruskal-Wallis *H* statistic) for analysis of the mechanism of action (MOA) of SAHA based on the ranking of correlation coefficients of the gene expression profiles inside and outside of pathways [21]. They applied the *H*-score method to the NCI60 microarray dataset (http://genome-www.stanford.edu/nci60/), found that gene expression coherence levels in pathways were significantly higher than in random gene sets, and confirmed that gene expression within a pathway tends to be coregulated and shared similar expression patterns [22]. Moreover, they found a significant difference in pathway gene expression coherence between tumor and normal cells under antitumor drug treatment [23]. We applied the *H*-score method to evaluate the pathway gene expression coherence of SAHA-treated tumor and normal samples. The *H*-score was calculated according to the following steps. For any pathway in the defined pathway set (here using *P*_*x*_ as example), the Pearson correlation coefficients between any gene pair within *P*_*x*_ were calculated. These values were called intrapathway *r* values. Then, Pearson correlation coefficients between any gene in *P*_*x*_ and any gene outside the pathway *P*_*x*_ in a defined pathway set were calculated. These values were called interpathway *r* values. The Pearson correlation coefficient (*r*) was defined as follows:

(1) $$\begin{array}{c} r=\frac{\sum_{i=1}^{n}\left(x_{i}-\overset{-}{x}\right)\left(y_{i}-\overset{-}{y}\right)}{\sqrt{\sum_{i=1}^{n}{\left(x_{i}-\overset{-}{x}\right)}^{2}\sum_{i=1}^{n}{\left(y_{i}-\overset{-}{y}\right)}^{2}}}, \end{array}$$

where *x*_*i*_ and *y*_*i*_ are a gene pair and $\overset{-}{x}$ and $\overset{-}{y}$ are average gene expression values of *x*_*i*_ and *y*_*i*_ over all samples (*n*). All Pearson correlation coefficients (interpathway and intrapathway) were ranked by their real values. Intrapathway and interpathway *r* value sets were used as two sample populations to calculate the *H*-score for any pathway as follows:

(2) $$\begin{array}{c} H=\frac{12}{N\left(N+1\right)}\overset{k}{\underset{t=1}{\sum }}\left(\frac{R_{t}^{2}}{n_{t}}\right)-3\left(N+1\right), \end{array}$$

where *R*_*t*_ is the rank sum of sample population *t*; *n*_*t*_ is the size of sample population *t*; *k* is the number of sample populations being compared (*k* = 2 in this study); and *N* = ∑_*t*=1_^*k*^ *n*_*t*_. Huang et al. found that when each of the *k* sample populations includes at least five observations, the sampling distribution of the *H*-score is a close approximation of the *χ* ^2^ distribution for *k* − 1 degrees of freedom [21]. To ascertain whether their conclusion was appropriate for our research, we conducted the following experiments. If a pathway had n genes, then n genes were randomly selected from the pool of total m genes (20,606 genes in our study) and the *H*-score was calculated. This procedure was repeated 1000 times for each pathway and the fraction of random *H*-scores larger than the *H*-score of the actual pathway was assigned as the *P* value of pathway coherence. Our results demonstrated that when a pathway included at least 15 genes and not more than 500 genes, the number of significantly cohesive (*P* < 0.05) pathways differed only slightly for the number obtained directly from the *χ* ^2^ distribution. Therefore, for accuracy and convenience, the *P* value of a pathway coherence was directly obtained using the *χ* ^2^ distribution with 1 degree of freedom (two-sample populations: *k* = 2 and *k* − 1 = 1; *P* < 0.05 equating *H*-score >3.84). A negative sign was assigned to the *H*-score if the middling rank of the intrapathway *r* values was lower than the rank of the interpathway *r* values. Therefore, a large positive *H*-score (>3.84) indicated a high level of gene expression coherence within the pathway compared to a random gene set.

### 2.4. Computational Method for Significant Changes in Pathway Coherence

To describe the change in pathway coherence state between tumor and normal samples, we defined two change states: up (specific high coherence in tumor samples but not in normal samples) and down (specific high coherence in normal samples but not in tumor samples). The statistical significance of up or down changes in pathway coherence were calculated as follows. If a pathway in the defined pathway set had n genes, a gene set (random pathway) was constructed by randomly selecting n genes from the pool of total m genes (20,606 genes in this study). The *H*-scores of the random pathway with tumor and normal gene expression profiles were calculated and compared. The proportions of up and down random pathways for a total of 1307 random pathways were counted. This procedure was repeated 1000 times and the background distribution of up and down pathways was determined. The fraction of the proportion of up or down random pathways that was greater than the proportion of the actual up or down pathways was assigned as the *P* value for up or down changes in pathway coherence. In evaluating the changes in pathway coherence at each time point, a total set of 24 samples was randomly permutated to generate 3 random tumor samples and 3 random normal samples. As described above, random pathways were built, the *H*-score of each random pathway with gene expression profiles in random tumor and normal samples was evaluated, and the *P* value for up or down changes in pathway coherence was calculated.

### 2.5. Cluster and Comparison of Time-Series Expression Data

Ernst et al. presented an algorithm specifically designed for clustering time-series expression data [24] and developed the STEM web-based program for analysis of time-series gene expression data [19]. STEM finds statistically significant patterns from time-series expression data and presents visual and interactive results with GO interpretation. We used STEM to cluster and compare time-series gene expression data from SAHA-treated tumor and normal samples. First, we defined a set of model profiles independent of the data representing distinct time-series gene expression patterns. The identification of significant model profiles was based on the hypergeometric test:

(3) $$\begin{array}{c} \overset{\min\left(m,s_{a}\right)}{\underset{i=v}{\sum }}\frac{\left(\begin{array}{c} m\\ i \end{array}\right)\left(\begin{array}{c} N-m\\ s_{a}-i \end{array}\right)}{\left(\begin{array}{c} N\\ s_{a} \end{array}\right)}, \end{array}$$

where *N* represents the number of total genes, *m* is the number of genes in the category of interest, *v* indicates the number of genes that are in the category of interest and are assigned to the model profile of interest, and *s*_*a*_ is the number of genes assigned to the model profile of interest. Significant model profiles were used to evaluate differential transcriptional responses in tumor and normal samples at different time points. Next, we identified model profile pairs by comparing gene expression data between tumor and normal samples and searching the intersection of the genes assigned to the two different profiles, one from the normal gene expression profiles and the other from the tumor gene expression profiles. This identified gene sets with consistent or distinct time-series expression patterns between tumor and normal samples. The significance of profile pairs was calculated as follows:

(4) $$\begin{array}{c} \overset{\min\left(n_{i},n_{j}\right)}{\underset{m=t}{\sum }}\frac{\left(\begin{array}{c} n_{j}\\ m \end{array}\right)\left(\begin{array}{c} N-n_{j}\\ n_{i}-m \end{array}\right)}{\left(\begin{array}{c} N\\ n_{i} \end{array}\right)}, \end{array}$$

where *N* represents the number of total genes, *n*_*i*_ is the number of genes with the profile *i* in the normal samples, *n*_*j*_ is the number of genes with the profile *j* in the tumor samples, and *t* is the number of the genes with the profile *i* in the normal samples and with the profile *j* in the tumor samples. Finally, we applied GO enrichment analysis to determine the biological significance of each gene set. GO terms with a corrected *P* value <0.05 were considered significant GO terms.

### 2.6. Identification and Pathway Mapping of Differentially Expressed Genes

Differential expression analysis of tumor and normal samples with SAHA treatment at different time points (0 h, 4 h, 12 h, and 24 h) was performed using GenMAPP software version 2 [25]. For all comparisons, genes were deemed significantly differentially expressed if the fold-change exceeded 1.5 and *P* < 0.05 (*t*-test). Genes significantly upregulated in tumor samples were defined as up; genes significantly upregulated in normal samples were defined as down. These genes were used to interpret time-specific transcriptional responses to the tumor-selective cytotoxic activity of SAHA. For each time point, differentially expressed genes were mapped to pathways using the MAPPFinder tool [26] to visually depict how SAHA differently regulated genes in pathways between tumor and normal samples at different time points.

## 3. Results and Discussion

### 3.1. Statistical Analysis of Pathway Coherence for Tumor and Normal Samples

**Figure 1:**
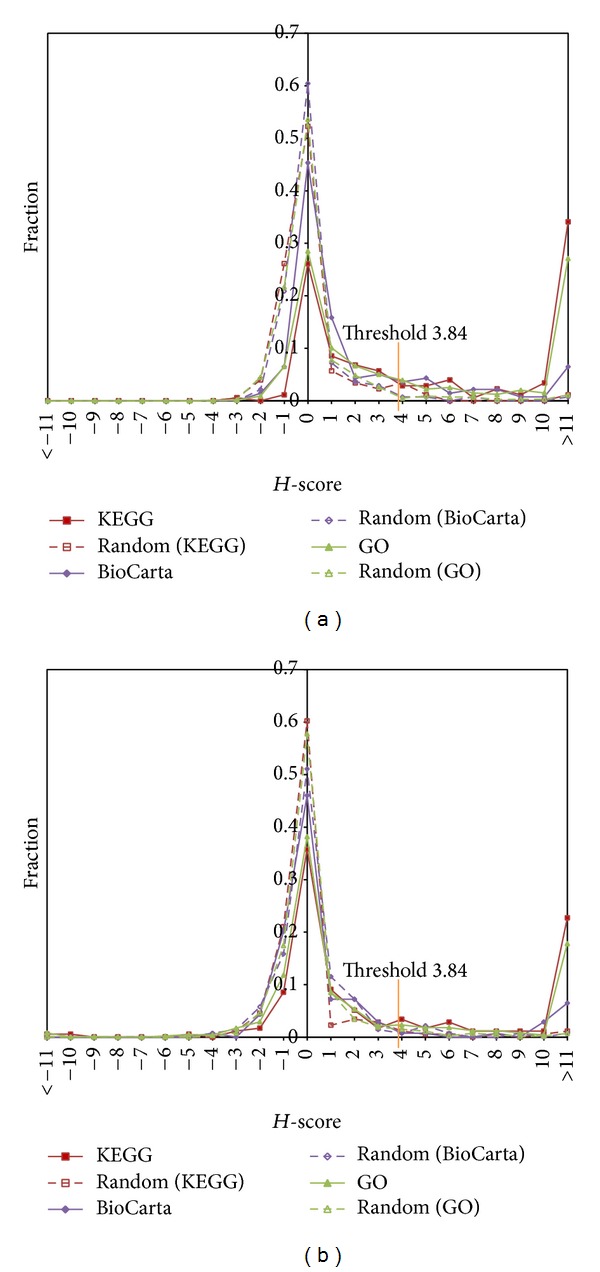
*H*-score distributions in KEGG, BioCarta, and GO pathways. (a) Mean *H*-score of pathways defined in KEGG (57.98, *P* = 4.41 × 10^−4^, *t*-test), BioCarta (3.05, *P* = 2.16 × 10^−4^, *t*-test), and GO (20.95, *P* = 1.51 × 10^−12^, *t*-test) for tumor samples. (b) Mean *H*-score of pathways defined in KEGG (38.07, *P* = 3.40 × 10^−12^, *t*-test), BioCarta, (2.39, *P* = 1.47 × 10^−12^, *t*-test), and GO (18.48, *P* = 1.91 × 10^−12^, *t*-test) for normal samples.

Pathway coherence is the coherence of its gene expression as measured by correlation strength. Genes in a pathway are believed to be regulated in a more coordinated fashion than a random pathway; thus, expression patterns of these genes are expected to be coherent. To test whether this hypothesis was appropriate for SAHA-treated tumor and normal samples, we computed *H*-score values for all defined pathways in KEGG, BioCarta, and GO. The detailed results of pathway *H*-score distributions are in Table S1 in Supplementary Materials available online at http://dx.doi.org/10.1155/2014/867289). *H*-score values of random gene sets (random pathways) with tumor and normal gene expression profiles were also evaluated. Figures 1(a) and 1(b) show distributions of the *H*-score values in the true pathways defined in KEGG, BioCarta, and GO and the responding random gene sets. Student's *t*-tests were performed to determine if *H*-score distributions between true pathways and the random pathways were significantly different. Computational results were mean *H*-scores of 57.98 for pathways defined in KEGG, 3.05 for BioCarta, and 20.95 GO for tumor gene expression profiles and 38.07 for KEGG, 2.39 for BioCarta, and 18.48 for GO for normal gene expression profiles. Corresponding random pathways were all close to 0 for both tumor and normal gene expression profiles. These differences were significant (see Figure 1).

If pathway coherence can be viewed as reflective of coordinated gene regulation, then changes in level can be viewed as a change in pathway regulation. We examined the extent to which individual pathways changed coherence, the types of pathways, and whether they achieved more or less coherence in tumor samples compared to normal samples. Using relative coherence states between tumor and normal samples, the 1307 pathways in the defined pathway set were divided into four categories: (1) consistently high (cohesive for both normal and tumor samples [227, 17.4%]); (2) up (cohesive only for tumor samples [296, 22.6%]); (3) down (cohesive only for normal samples [114, 8.7%]); and (4) consistent low (not cohesive for tumor or normal samples [670, 51.3%]). This finding implied that most pathways (17.4% + 51.3% = 68.7%) had unchanged coherence, while a few pathways (22.6% + 8.7% = 31.3%) showed a significant change in coherence when tumor and normal samples were compared (*P* < 0.001). Moreover, the proportion of up pathways was significantly higher than down pathways (22.6% versus 8.7%; *P* = 2.38 × 10^−14^, Fisher's exact test), suggesting that the pathway with tumor samples tended to be more coherent than the pathway with normal samples. We further analyzed the coherence changes in different functional categories defined in KEGG and BioCarta. For each category, the number of up and down pathways was compared and a Fisher's exact test determined if detected differences were statistically significant. Up pathways were found in metabolism (29.2% versus 6.6%) and organismal systems (28.6% versus 5.7%) categories in KEGG with significance levels <0.05. Down pathways were found in cell cycle regulation (0% versus 55.6%) in BioCarta with significance levels <0.05 (Table 1). Grayson et al. reported that HDAC inhibitors altered expression patterns of mRNAs and proteins associated with cell cycle arrest [27]. Our results confirmed that pathways related to the cell cycle might be important in SAHA induction of tumor-selective apoptotic responses.

Statistical analysis of pathway coherence at each time point was performed for tumor and normal samples. Similar to results described above, true pathways were found to be significantly more coherent than random gene sets at all time points (0 h, 4 h, 12 h, and 24 h) for both tumor and normal samples (Table 2). Several pathways showed consistent coherence across time series for both tumor and normal samples, such as the GO category structural constituent of ribosome and KEGG category ribosome. However, some pathways changed coherence states across time series. We conjectured that these pathways might be involved in SAHA-induced tumor-specific responses. We found that 667 pathways exhibited variation in coherence across time series for tumor samples following SAHA treatment. Only 480 pathways showed variation in coherence for normal samples. This result suggested that pathways changed coherence states in tumor samples (51.0% versus 36.7%; *P* = 5.57 × 10^−11^, Fisher's exact test). By investigating time-specific up and down pathways, we found that about 30% of pathways showed significant coherence changes between tumors and normal samples at each time point. For example, the DNA repair, cell cycle, p53, and mitochondrial respiratory chain pathways all showed a coherence change between tumor and normal samples at some time points. Moreover, proportions of up and down pathways varied across the time series. Compared to down pathways the proportion of up pathways was significantly higher at 0 h and 24 h (0 h, 16.4% versus 13.2%; *P* = 1.18 × 10^−2^; 24 h; 20.0% versus 7.0%; *P* = 2.84 × 10^−14^, Fisher's exact test) and lower at 4 h (9.2% versus 14.5%; *P* = 3.75 × 10^−5^, Fisher's exact test), with no significant difference at 12 h (14.2% versus 16.2%; *P* = 0.16, Fisher's exact test).

### 3.2. Identification of Pathway Coherence Variation Patterns in Time Series

If 1 represents the coherence state and 0 represents the noncoherence state at a certain time point, the TVPC pattern 0001 represents a pathway with noncoherence at 0 h, 4 h, and 12 h and with coherence at 24 h. Our results found that patterns 1000, 0001, 1001, 0101, and 1011 were significantly enriched for tumor samples and patterns 0000, 1100, 1110, and 1111 were significantly enriched for normal samples (*P* < 0.05, Fisher's exact test). We constructed 256 combination TVPC patterns for investigating the correlation of coherence variation between tumor and normal pathways. For instance, the pattern 1111 versus 0000 represented high coherence of a pathway in tumor samples and low coherence of the pathway in normal samples. Of the combination patterns, 43.6% (109/256) did not contain any pathways, 19.6% (50/256) contained only a single pathway, and 12.5% (32/256) contained more than 6 pathways. These results showed that about 80% of pathways belonged to a few combination patterns. Therefore, the influence of SAHA on tumor and normal cells was not chaotic but organized and subject to control. Based on extensive analysis of the correlation between combination patterns and pathway functions, we devised a grouping tactic that divided all combination patterns into five categories: (1) no response to SAHA (426 pathways within 4 patterns); (2) consistent response to SAHA with the same variation pattern for both tumor and normal samples (26 pathways within 8 patterns); (3) divergent response to SAHA for tumor and normal samples (240 pathways within 88 patterns); (4) tumor-specific response to SAHA for only tumor samples (401 pathways within 22 patterns); and (5) normal specific response to SAHA for only normal samples (214 pathways within 25 patterns). Detailed descriptions of pathways belonging to each TVPC pattern and combination TVPC pattern are in Tables S2–S6. The no response and consistent response both related to fundamental biological functions. For example, the GO term structural constituent of ribosome and the KEGG term ribosome belonged to the no response category (1111 versus 1111), and the GO terms ribosome, organellar ribosome, and mitochondrial ribosome belonged to the consistent response category (0111 versus 0111). SAHA treatment had no effect on these pathways. In contrast, the combination patterns divergent response, tumor-specific response, and normal specific response involving DNA damage response, cell cycle, and mitochondrion integrity pathways might be involved in differential regulation between tumor and normal samples.

Table S4 (tumor-specific response) shows that the GO terms DNA metabolic process, response to DNA damage stimulus, and DNA repair and the KEGG pathways DNA replication and mismatch repair all belonged to the pattern 1000 versus 1111 and are involved in the DDR. These results indicated that from 0 h to 4 h, SAHA treatment of tumor samples irreversibly reduced the coherence of gene expression in pathways related to DDR. Bakkenist and Kastan reported that HDAC inhibitors activate the DDR [28]. Groselj et al. reported that HADC inhibitors are radiosensitisers with large effects on DNA damage and repair [29]. Robert and Rassool reported HDAC inhibitors trigger widespread histone acetylation and increases in DNA damage and induction of apoptosis in tumor cells [30]. Our results demonstrated that SAHA immediately inhibited the DNA repair process at an early stage of drug treatment, while showing no effect on normal samples.

Table S5 (normal specific response) shows that the GO term mitotic cell cycle checkpoint belongs to the pattern 0000 versus 1100, the GO terms DNA integrity checkpoint and DNA damage checkpoint belong to the pattern 0000 versus 0100, and the GO term cell cycle checkpoint belongs to the pattern 0000 versus 1110. These results indicated that pathways related to cell cycle checkpoint lost expression coherence for tumor samples with SAHA treatment. Furthermore, Table S6 (divergent response) shows that the GO terms cell cycle process, cell cycle phase, mitotic cell cycle, M phase, M phase of mitotic cell cycle, mitotic, and spindle all belonged to the pattern 0010 vesus 1110. With SAHA treatment, cell cycle-related pathways showed a time-specific expression coherence for tumor samples at 12 h, while maintaining high coherence from 0 h to 12 h for normal samples. Therefore we hypothesized that, for tumor samples, SAHA treatment led to DNA damage accumulation and inhibition of cell cycle checkpoints. Cells could not enter cell cycle correctly and thus DNA damage could not be correctly repaired.

Table S6 (divergent response) shows that the KEGG p53 signalling pathway belonged to pattern 0101 versus 0111 and the BioCarta p53 hypoxia pathway belonged to 1001 versus 0010. Activation of p53 is closely related to DNA damage and cell cycle arrest. Loewer et al. reported that p53 activation is linearly correlated with degree of DNA damage [31]. In addition to its well-studied role in cell cycle checkpoints, the p53 pathway induces apoptosis in cancer cells [32]. Cao et al. reported that the antiparasitic clioquinol is a HDAC inhibitor that induces expression of p53, leading to apoptosis in leukemia and myeloma cells [33]. Wu et al. reported that p53 mediates reduced expression of Bcl-2 in human hepatoma HepG2 cells [34]. Aoyama et al. reported that activation of p53 increases Bax expression but maintains lower expression of Bcl-2 [35]. Also from Table S6 (divergent response), GO terms apoptosis and programmed cell death both belonged to 1001 versus 0010. With SAHA treatment, apoptosis-related pathways showed time-specific expression coherence at 0 h and 24 h for tumor samples. Moreover, Table S4 (tumor-specific response) shows that pathways related to mitochondrial integrity such as mitochondrial envelope, mitochondrial membrane, mitochondrial inner membrane, and mitochondrial respiratory chain belong to the pattern 0001 versus 1111. These findings demonstrated that SAHA treatment causes morphological and physiological alterations of mitochondrial integrity at 24 h in tumor samples. Mitochondrial integrity is maintained by prosurvival Bcl-2 family proteins, while proapoptotic BH3-only proteins function as sensors of cellular stress and activate the intrinsic apoptosis pathway [36]. Rikiishi reported that HDAC inhibitors downregulate prosurvival proteins such as Bcl-2 and upregulate proapoptotic proteins such as Bim, Bak, and Bax [37]. Therefore, we hypothesized that SAHA treatment of tumor samples, through direct or indirect actions, activated the p53 pathway, influencing the expression of Bcl-2 family genes and mitochondrial integrity and contributing to tumor cell apoptosis.

### 3.3. Clustering Analysis of Time-Series Gene Expression Data

**Figure 2:**
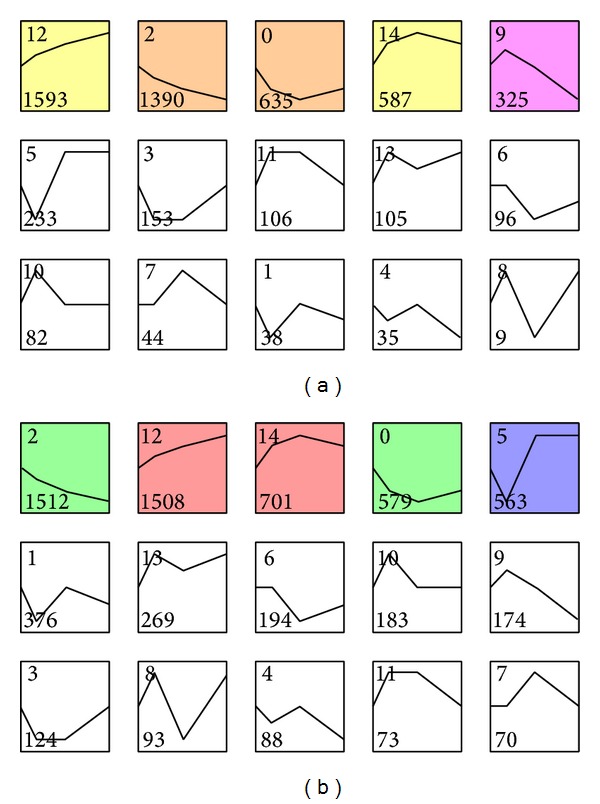
Gene expression profiles for normal and tumor samples. (a) Gene expression model profiles for normal samples. (b) Gene expression model profiles for tumor samples. Each box corresponds to a preestablished expression profile (number 1 to number 15), sorted according to the number of genes in each profile. Colored profiles have statistically significant enrichment.

We used STEM [19] to cluster time-series gene expression data. We chose 15 preestablished expression profiles representing various gene expression patterns across time-series and significant expression profiles for tumor and normal samples that were identified. We found that 5 profiles (numbers 0, 2, 9, 12, and 14) were significantly enriched for normal samples and 5 (numbers 0, 2, 5, 12, and 14) were significantly enriched for tumor samples (Figure 2). Profiles numbers 0, 2, 12, and 14 were shared among tumor and normal samples. Among the common profiles, numbers 2 and 12 were the most significant and they represent two antipodal gene expression patterns. Profile number 2 showed a gradual decline in expression from 0 h to 24 h, whereas profile number 12 showed a gradual increase in expression from 0 h to 24 h (Figures 2(a) and 2(b)). Similarly, profiles numbers 0 and 14 show two antipodal gene expression patterns. Profile number 0 has declined expression from 0 h to 12 h and increased expression from 12 h to 24 h; profile number 14 has the opposite expression pattern. Profile number 5 is enriched in tumor samples and number 9 is enriched in normal samples. Profile number 5 declined from 0 h to 4 h, increased from 4 h to 12 h and was unchanged from 12 h to 24 h. Profile number 9 increased from 0 h to 4 h and declined from 4 h to 24 h. A complete description of genes for each profile is in Tables S7–S16. For example, genes GATA and KLF belong to profile number 2 and the gene GLRX is in profile number 12. Akada et al. reported that expression of GATA and KLF is significantly downregulated by SAHA treatment in human erythroleukemia cells [38]. Glaser et al. reported that SAHA produced a dose-dependent increase in gene expression of GLRX [39].

### 3.4. Biological Function Discovery from STEM Profile Pairs

**Figure 3:**
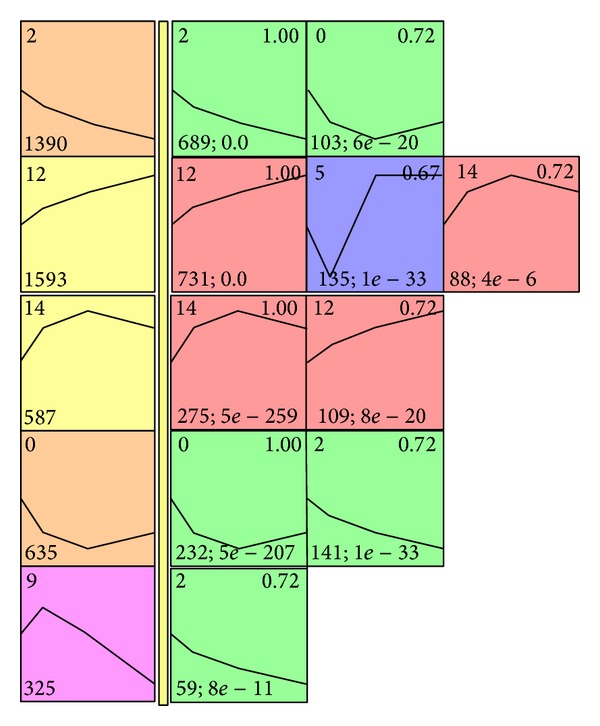
Significant expression profile pairs between normal and tumor samples. On left side, yellow bar, profiles are significantly enriched for normal samples. On right side, yellow bar, profiles are significantly enriched for tumor samples with a significant intersection with the leftmost profile in the row. Profile pairs are sorted by statistical significance of intersection.

Using the comparison function of STEM, we extracted 10 significant profile pairs between tumor and normal samples [19]. The 10 profile pairs can be divided into three categories: (1) invariant (genes within the profile pairs show the same expression patterns for tumor and normal samples); (2) minitrim (genes within the profile pairs show a small difference in expression profiles at a certain time point between tumor and normal samples, for example, the profile pair numbers 0 and 2); (3) reversion (genes within the profile pairs show an antipodal gene expression pattern at some time points between tumor and normal samples, for example, the profile pair numbers 9 and 2) (Figure 3). Simply excavating gene lists of each profile pair did not allow interpretation of the SAHA MOA. A popular approach to gaining biological insights from a set of identified genes is to identify the GO terms annotations that are overrepresented among genes in the set. Therefore, GO enrichment analyses were conducted for gene sets corresponding to each profile pair. A brief description of GO terms enriched in each profile pair is in Table 3. A complete description of GO terms within each profile pair is in Tables S17–S19.

GO terms related to protein acetylation modification belong to the invariant profile pair numbers 0 and 0 such as protein acetylation, histone acetylation, histone acetyltransferase activity, and acetyltransferase complex (Table 3). SAHA is a pan-inhibitor of many kinds of HDACs and provokes a broad spectrum of cellular acetylation modification levels [40]. Our results demonstrated that SAHA treatment downregulated expression of genes related to acetylation modification from 0 h to 12 h and then upregulate these genes from 12 h to 24 h. Moreover, GO terms related to ribonucleoside triphosphates such as ribonucleoside triphosphate metabolic process, ribonucleoside triphosphate catabolic process, and ribonucleoside metabolic process were significantly enriched in the invariant profile pair numbers 14 and 14. These GO terms represent chemical reactions and pathways involving ribonucleoside triphosphates. Genes related to these fundamental biological functions maintained the same expression profile in tumor and normal samples and might not be involved with SAHA-induced, tumor cell-selective apoptotic responses. Many GO terms related to DDR belonged to the minitrim profile pair numbers 2 and 0 such as DNA-dependent DNA replication, DNA metabolic process, DNA repair, and cellular response to DNA damage stimulus. Our results indicated that SAHA treatment gradually downregulated DDR-related genes from 0 h to 24 h for normal samples and downregulated them from 0 h to 12 h and then upregulated them from 12 h to 24 h in tumor samples. SAHA might differently regulate DDR-related genes, which confirms previous observations. Our results reflected the cardinal distinction between DNA stability-maintaining mechanisms in tumor and normal samples treated with SAHA that led to responses of downstream pathways. GO terms related to the cell cycle belonged to the reversion profile pair numbers 9 and 2, such as spindle organization, cell cycle, mitosis, and cell cycle process. This indicated that SAHA treatment gradually downregulated cell cycle-related genes from 0 h to 24 h for tumor samples and upregulated these genes from 0 h to 4 h and then downregulated them from 4 h to 24 h for normal samples. STEM analysis indicated different effects of SAHA treatment on cell cycle-related pathways. The GO term mitochondrion belonged to the reversion profile pair numbers 12 and 5. SAHA treatment gradually upregulated genes related to mitochondria from 0 h to 24 h in normal samples and downregulated these genes from 0 h to 4 h and then upregulated them from 4 h to 24 h in tumor samples. Mitochondrial integrity is maintained by pro-survival Bcl-2 family proteins, while pro-apoptotic BH3-only proteins function as cellular stress sensors and activate the intrinsic apoptosis pathway [36]. Therefore, SAHA might disrupt mitochondrial integrity, inducing intrinsic apoptosis. In summary, minitrim and reversion profile pairs were closely related to DDR, cell cycle, and mitochondrial integrity processes. We propose that SAHA differently regulated DDR-related genes between tumor and normal samples and inhibited cell cycle checkpoint functions in tumor samples, disrupting mitochondrial integrity and leading to cell apoptosis.

### 3.5. Determining the Tumor Cell-Selective, Proapoptotic Mechanism of SAHA

**Figure 4:**
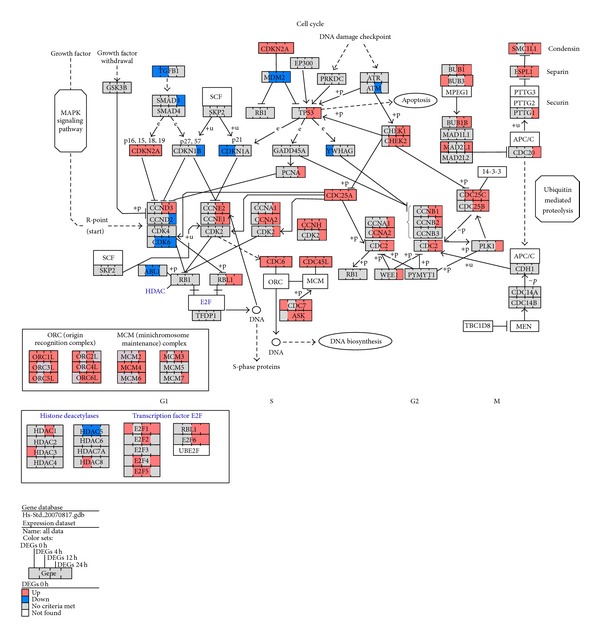
Timepoint comparisons of cell cycle genes differently expressed between tumor and normal samples with SAHA treatment. Red, up: highly expressed in tumor samples (>1.5 fold). Blue, down: highly expressed in normal samples (>1.5 fold). Grey: no criteria were met. White: not in the dataset.

Results from TVPC and STEM analyses indicated that SAHA treatment induced differential responses in DNA damage-related pathways. We examined the expression profiles of DNA damage-related genes and found that RAD9, PARP1, BRCA1, CHK1, CHK2 (RAD53), and CHEK2 had high expression in SAHA-treated tumor samples. Broustas and Lieberman reported that several genes including RAD9, PARP1, and BRCA1 participate in DDR and are vital for the maintenance of genome stability [41]. RAD9 encodes an adaptor protein with a well-characterized function in the DNA damage checkpoint response [42]. PARP1 encodes a chromatin-associated enzyme, poly (ADP-ribosyl) transferase, which is dependent on DNA and involved in recovery from DNA damage [43]. BRCA1 encodes a nuclear phosphoprotein that is important in transcription, DNA repair of double-stranded breaks, and recombination [44]. Heideker et al. reported that DDR activation relies on the activity of Ser/Thr kinases such as CHK1 and CHK2 (RAD53) [45]. CHEK2 contains a forkhead-associated protein interaction domain essential for activation in response to DNA damage. The domain is rapidly phosphorylated in response to replication blocks and DNA damage [46]. Based on the above pathway-scale and gene-scale analysis, we infer that SAHA treatment might cause multiform DNA damage in tumor samples and could activate downstream signal pathways responding to accumulation of DNA damage. In pathway-scale analysis, by integrating two aspects of results from TVPC and STEM analyses, we deduced that SAHA treatment differentially regulated gene expression profiles of pathways related to p53, cell cycle, mitochondrial integrity, and cell apoptosis (pathway-scale analysis). The p53 pathway is the core signalling pathway that mediates interactions among these biological process following intrinsic apoptosis. Therefore, in gene-scale analysis, we further investigated expression of genes related to p53, cell cycle, and cell apoptosis pathways in tumor and normal samples. Using GenMAPP [26], we identified differentially expressed genes for each time point and mapped them to the cell cycle and cell apoptosis pathways.  Figure 4 shows multiple time point comparisons of genes differently expressed between tumor and normal samples in the cell cycle pathway. ATR was not differently expressed and ATM was downregulated at 12 h; p53 was upregulated at 12 h in tumor samples. P53 is a tumor suppressor protein containing transcriptional activation, DNA binding, and oligomerization domains that respond to diverse cellular stresses to regulate expression of target genes and can induce cell cycle arrest, DNA repair, and apoptosis [47]. ATM belongs to the PI3/PI4-kinase family and regulates a wide variety of downstream proteins including p53. ATM and the closely related kinase ATR are master controllers of cell cycle checkpoint pathways required for cell response to DNA damage and for genome stability [48, 49]. However, we found that ATR and ATM are not activated and thus do not initiate p53 activation in tumor samples. Kotsinas et al. reported that CDKN2A (ARF) mediates p53 activation with the DDR axis in addition to the ATM/ATR pathway [50]. ARF stabilizes the tumor suppressor protein p53, which interacts with the E3 ubiquitin-protein ligase MDM2, which is responsible for p53 degradation [51, 52]. ARF had consistently high expression, while MDM2 was downregulated after 4 h (Figure 4). Therefore, ARF and MDM2 might be one of the prime reasons for p53 activation in tumor samples after 12 h of SAHA treatment. We further found that CCNE1 (cyclin E1), CCNE2 (cyclin E2), and CDK2 were all upregulated, while CDKN1A (P21) and CDKN1B (P27) were downregulated in tumor samples. Cyclins E1 and E2 belong to the highly conserved cyclin family, form complexes with CDK2, and function as regulatory subunits of CDK2. The cyclin E-CDK2 complexes are required for the cell cycle G1/S transition. They accumulate at the G1-S phase boundary and are degraded as cells progress through S phase [53–55]. P21 and p27 are cyclin-dependent kinase inhibitors. They bind to and inhibit cyclin E-CDK2 complexes and thus function as regulators of G1 cell cycle progression. P53 induces high expression of p21 and p27 and mediate p53-dependent cell cycle G1 arrest in response to accumulation of DNA damage [56, 57]. However, activation of p53 did not lead to high expression of p21 and p27 and thus, formation of cyclin E-CDK2 complexes were not inhibited effectively (Figure 4). Cyclin E-CDK2 complexes activate E2F family proteins by interaction with the retinoblastoma protein [58]. Massip et al. reported that E2F1 activates p53 transcription and participates in apoptosis induction [59]. In summary, we propose that DNA damage accumulation in tumor samples activated p53 but did not lead to cell cycle arrest because of loss of function of the DNA damage checkpoint. Excessive formation of cyclin E-CDK2 complexes induced high expression of the E2F family such as E2F1 and E2F2, resulting in further p53 activation. This resulted in positive feedback regulation of p53.

**Figure 5:**
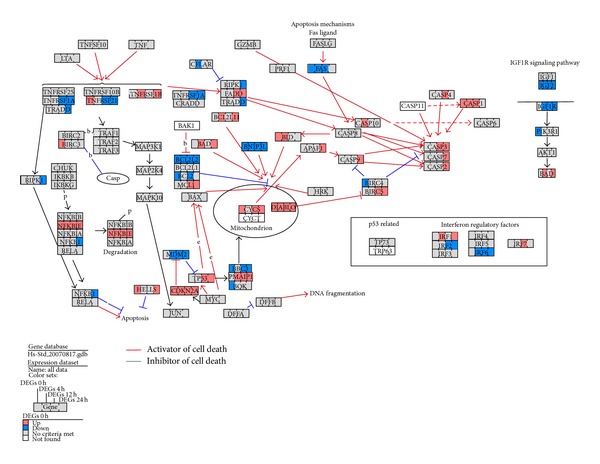
Timepoint comparisons of apoptosis genes differently expressed between tumor and normal samples with SAHA treatment. Red, up: highly expressed in tumor samples (>1.5 fold). Blue, down: highly expressed in normal samples (>1.5 fold). Grey: no criteria were met. White: not in dataset.

Figure 5 shows multiple timepoint comparisons of differently expressed apoptosis genes between tumor and normal samples. After 24 h of SAHA treatment, executor caspase-2, caspase-3, and caspase-7 were specifically highly expressed in tumor samples, indicating initiation of irreversible apoptosis. Caspase-8 and death receptors FAS and TNFR were not highly expressed, whereas genes related to intrinsic apoptosis such as cytochrome C (CYCS), Smac (DIABLO), caspase-9, and Apaf-1 were significantly highly expressed. This result indicated that apoptosis response in tumor samples was initiated through the intrinsic but not the extrinsic apoptosis pathway. We further observed that Bcl-2 family proteins were differently expressed between tumor and normal samples. Antiapoptotic Bcl-2 family proteins such as Bcl-2 and BCL2L2 were downregulated in tumor samples, while proapoptotic Bcl-2 family proteins such as Bad and BCL2L11 were upregulated. Thompson et al. reported that the expression of Bcl-2 and Bim in cancer cell lines confers further resistance or sensitivity, respectively, to HDAC inhibitor treatment [60]. We propose that SAHA treatment resulted in the activation of p53 we described. P53 is an inhibitor of antiapoptotic Bcl-2 family proteins and an activator of proapoptotic Bcl-2 family proteins. P53 activation altered the balance between antiapoptotic and proapoptotic proteins, resulting in mitochondrial outer membrane permeabilization (MOMP) and irreversible intrinsic apoptosis in SAHA-treated tumor samples.

**Figure 6:**
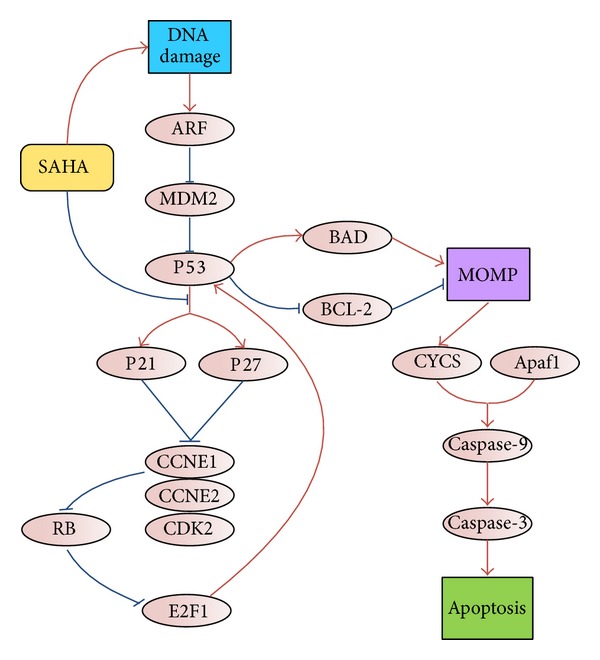
Deductive regulation model of SAHA-induced tumor cell-selective apoptosis.

Based on the above analyses, we propose a possible regulation model of tumor cell-selective apoptosis induced by SAHA (Figure 6). In tumor samples, accumulation of  DNA damage due to SAHA treatment activates p53 by upregulating ARF and downregulating MDM2. P21 and p27 are downregulated so they do not mediate cell cycle arrest following p53 activation. Downregulated p21 and p27 do not inhibit cyclin E1 and cyclin E2 from forming cyclin E-CDK2 complexes with CDK2. The cyclin E-CDK2 complexes activate E2F1 by interaction with RB. E2F1 contributes to p53 activation following the G1-to-S transition. Activated p53 downregulates antiapoptotic Bcl-2 family proteins (e.g., Bcl-2) and upregulates proapoptotic Bcl-2 family proteins (e.g., Bad), altering the balance between antiapoptotic and proapoptotic proteins, inducing MOMP. MOMP causes mitochondrial CYCS (cytochrome C) release into the cytoplasm to form complexes with caspase-9 and Apaf-1 and further activate executer caspases (e.g., caspase-3). These processes lead to irreversible intrinsic apoptosis.

## 4. Conclusions

We provide a novel analysis framework for time-series gene expression profiles to elucidate the mechanism of tumor cell-selective proapoptotic responses induced by SAHA. Pathway-scale analyses by TVPC and STEM identified pathways with a significant change in coherence level and profile patterns between tumor and normal samples. In particular, pathways related to DNA damage response, DNA repair, cell cycle, p53, mitochondrial respiratory chain, mitochondrial integrity, and apoptosis were significantly enriched. Gene-scale analysis by GenMAPP identified pathways related to DNA damage response, cell cycle, p53, and mitochondrial integrity with a direct impact on tumor cell-selective apoptosis induced by SAHA. Based on our results, a possible regulation model of SAHA-induced tumor-selective apoptosis was proposed. Our research extends the original research of Bolden et al. The proposed analysing framework for time-series gene expression profiles is operable and extensible for explaining the MOA of anticancer drugs.

## Supplementary Material

Table S1. H-score of all pathways for tumor and normal samples: This table lists the name, category, website and H-score values for all 1307 pathway.
Table S2. Illustrative pathways belonging to term "No responding": This table lists the name, category, website and TVPC patterns for "No responding" pathways.
Table S3. Illustrative pathways belonging to term "Consistent responding": This table lists the name, category, website and TVPC patterns for "Consistent responding" pathways.
Table S4. Illustrative pathways belonging to term "Tumor specific responding": This table lists the name, category, website and TVPC patterns for "Tumor specific responding" pathways.
Table S5. Illustrative pathways belonging to term "Normal specific responding": This table lists the name, category, web site and TVPC patterns for "Normal specific responding" pathways.
Table S6. Illustrative pathways belonging to term "Divergent responding": This table lists the name, category, website and TVPC patterns for "Divergent responding" pathways.
Table S7. Genes within profile #0 for normal samples: This table contains all genes within profile #0 for normal samples, and lists the normalized expression data to the right of gene symbol.
Table S8. Genes within profile #2 for normal samples: This table contains all genes within profile #2 for normal samples, and lists the normalized expression data to the right of gene symbol.
Table S9. Genes within profile #9 for normal samples: This table contains all genes within profile #9 for normal samples, and lists the normalized expression data to the right of gene symbol.
Table S10. Genes within profile #12 for normal samples: This table contains all genes within profile # 12 for normal samples, and lists the normalized expression data to the right of gene symbol.
Table S11. Genes within profile #14 for normal samples: This table contains all genes within profile #14 for normal samples, and lists the normalized expression data to the right of gene symbol.
Table S12. Genes within profile #0 for tumor samples: This table contains all genes within profile #0 for tumor samples, and lists the normalized expression data to the right of gene symbol.
Table S13. Genes within profile #2 for tumor samples: This table contains all genes within profile #2 for tumor samples, and lists the normalized expression data to the right of gene symbol.
Table S14. Genes within profile #5 for tumor samples: This table contains all genes within profile #5 for tumor samples, and lists the normalized expression data to the right of gene symbol.
Table S15. Genes within profile #12 for tumor samples: This table contains all genes within profile #12 for tumor samples, and lists the normalized expression data to the right of gene symbol.
Table S16. Genes within profile #14 for tumor samples: This table contains all genes within profile #14 for tumor samples, and lists the normalized expression data to the right of gene symbol.
Table S17. GO terms enriched for "Invariant" profile pairs: This table lists the ID, name, P-value and corrected P-value for GO terms enriched for "Invariant" profile pairs.
Table S18. GO terms enriched for "Minitrim" profile pairs: This table lists the ID, name, P-value and corrected P-value for GO terms enriched for "Minitrim" profile pairs.
Table S19. GO terms enriched for "Reversion" profile pairs: This table lists the ID, name, P-value and corrected P-value for GO terms enriched for "Reversion" profile pairs.

## Figures and Tables

**Table 1:** Pathway coherence changes by functional category.

Pathway category	Pathway count	Consistent high	Up	Down	Consistent low	*P* (up versus down)
KEGG						
Cellular processes	12	50	8.3	16.7	25	1
Environmental information processing	16	18.8	37.5	12.5	31.3	0.22
Genetic information processing	15	53.3	33.3	6.7	6.7	0.17
Human diseases	37	24.3	13.5	13.5	48.6	1
** Metabolism**	61	23	29.5	6.6	41	**0.017**
** Organismal systems**	35	11.4	28.6	5.7	54.3	**0.02**
BioCarta						
Adhesion	11	27.3	9.1	0	63.6	1
Apoptosis	22	4.5	13.6	4.5	77.3	0.61
Cell activation	14	0	14.3	0	85.7	0.48
** Cell cycle regulation**	9	0	0	55.6	44.4	**0.03**
Cell signalling	106	3.8	16	6.6	73.6	0.11
Cytokinesis/chemokines	34	2.9	8.8	2.9	85.3	0.61
Developmental biology	11	9.1	18.2	0	81.8	0.48
Expression	15	0	33.3	20	46.7	0.68
Hematopoiesis	10	0	30	0	70	0.21
Immunology	39	0	10.3	0	89.7	0.12
Metabolism	2	50	0	0	50	1
Neuroscience	6	0	50	0	50	0.18

**Table 2:** Coherence levels as average *H*-score between true and random pathways at different time points.

	True	Random	*P* value (Student's *t*-test)
Tumor (0 h)	6.97	0.42	1.03 × 10^−14^
Tumor (4 h)	4.09	0.39	1.89 × 10^−12^
Tumor (12 h)	4.76	0.51	1.26 × 10^−09^
Tumor (24 h)	11.87	0.58	3.81 × 10^−09^
Normal (0 h)	11.61	0.48	5.89 × 10^−08^
Normal (4 h)	10.44	0.44	3.88 × 10^−15^
Normal (12 h)	17.60	0.40	2.72 × 10^−07^
Normal (24 h)	6.83	0.44	1.80 × 10^−04^

**Table 3:** Gene functional enrichment of GO terms in profile pairs (*P* < 0.05).

Profile pair (number of genes)	GO terms	*P* value	Count	GO terms	*P* value	Count
**“Invariant”**						
#0-#0 (232)	Cellular macromolecule metabolic process	1.50*E* − 13	128	RNA metabolic process nucleic acid binding	2.30*E* − 13	90
	GO:0034645			GO:0016070		
	Nucleic acid metabolic process	1.80*E* − 13	98	Nucleic acid binding	7.50*E* − 12	83
	GO:0090304			GO:0003676		
	Nucleus GO:0005634	2.20*E* − 13	111	RNA metabolic process GO:0016070	5.35*E* − 06	130
#2-#2 (689)	Protein binding GO:0005515	5.50*E* − 14	461	Intracellular membrane-bounded organelle GO:0043231	1.40*E* − 09	371
	Intracellular part GO:0044424	1.50*E* − 10	464	Single-organism cellular process GO:0044763	1.20*E* − 09	74
	Membrane-bounded organelle GO:0043227	4.00*E* − 10	401	Intracellular organelle GO:0043229	1.70*E* − 09	419
	Response to stress GO:0006950	5.90*E* − 10	167			
#12-#12 (731)	Cytoplasm GO:0005737	1.7*E* − 12	397	Oxidoreductase activity GO:0016491	1.70*E* − 09	404
	Carboxylic acid metabolic process GO:0019752	8.60*E* − 11	71	Cofactor metabolic process GO:0051186	3.80*E* − 08	29
	Small molecule metabolic process GO:0044281	7.50*E* − 10	158	Carboxylic acid catabolic process GO:0046395	1.60*E* − 07	24
	Oxoacid metabolic process GO:0043436	1.20*E* − 09	74	Intraciliary transport particle GO:0030990	3.40*E* − 07	7
#14-#14 (275)	Ribonucleoside triphosphate metabolic process	1.00*E* − 05	24	Ribonucleoside triphosphate catabolic process	2.50*E* − 05	22
	GO:0009199			GO:0009203		
	Nucleoside triphosphate metabolic process GO:0009141	1.60*E* − 05	24	Ribonucleoside metabolic process GO:0009119	3.00*E* − 05	25

**“Minitrim”**						
#2-#0 (103)	Negative regulation of RNA metabolic process GO:0051253	4.60*E* − 10	25	Regulation of RNA metabolic process GO:0051252	6.80*E* − 08	45
	Negative regulation of gene expression GO:0010629	1.20*E* − 08	25	Gene expression GO:0010467	3.30*E* − 07	60
	Negative regulation of biosynthetic process GO:0009890	1.20*E* − 08	26	Regulation of transcription, DNA-templated GO:0006355	4.90*E* − 07	42
#0-#2 (141)	DNA-dependent DNA replication GO:0006261	1.70*E* − 06	7	DNA replication GO:0006260	1.50*E* − 05	9
	DNA metabolic GO:0006259	3.50*E* − 06	17	Cellular response to DNA damage stimulus GO:0000278	3.70*E* − 05	13
	Replication fork GO:0005657	5.00*E* − 06	5	DNA repair GO:0006281	4.60*E* − 05	10

**“Reversion”**						
#9-#2 (59)	Spindle organization GO:0007051	7.30*E* − 09	7	Mitotic cell cycle GO:0000278	4.50*E* − 07	13
	Cell division GO:0051301	8.80*E* − 09	14	Cell cycle GO:0007049	3.00*E* − 06	16
	Nuclear division GO:0000280	1.10*E* − 08	12	Regulation of nuclear division GO:0051783	4.40*E* − 05	5
	Mitosis GO:0007067	6.90*E* − 08	10	Regulation of cell cycle GO:0051726	5.20*E* − 05	10
	Cell cycle process GO:0022402	4.50*E* − 07	15	Regulation of mitotic cell cycle GO:0007346	6.70*E* − 05	7
#12-#5 (135)	Mitochondrion GO:0005739	1.20*E* − 05	24			

The number of overlapped genes of each “invariant,” “minitrim,” and “reversion” profile pairs is listed in the bracket below the name of each profile pairs in this table.
